# Remote ischemic preconditioning fails to enhance maximal accumulated oxygen deficit in well-trained college tennis players

**DOI:** 10.7717/peerj.20789

**Published:** 2026-02-06

**Authors:** Xinshi Zhao, Yongji Yang, Yameng Wang

**Affiliations:** 1 Department of Physical Education, North China University of Water Resources and Electric Power, Zhengzhou, China; 2 College of Physical Education and Health, East China Jiaotong University, Nanchang, China

**Keywords:** Maximal accumulated oxygen deficit, Oxygen uptake, Blood lactate, Excess post-exercise oxygen consumption

## Abstract

**Background:**

Tennis, characterized by intermittent high-intensity bursts, demands both aerobic and anaerobic energy pathways for optimal performance. Remote ischemic preconditioning (RIPC) has emerged as a potential method to enhance athletic outcomes. This study aimed to explore the impact of RIPC on anaerobic capacity, specifically the maximal accumulated oxygen deficit (MAOD), in well-trained college tennis players.

**Methods:**

In a single-blinded, randomized, controlled crossover design, 16 participants (eight men and eight women; age: 20.9 ± 1.4 years; height: 1.73 ± 0.76 m; weight: 63.5 ± 8.2 kg; BMI: 21.2 ± 1.4 kg/m^2^) completed supramaximal intensity tests across baseline, placebo, and RIPC conditions. RIPC involved alternating bilateral occlusion of 220 or 20 mmHg for 4 × 5 min applied to both arms. Subsequently, the subjects performed a supramaximal test on the treadmill at 110% VO_2max_ intensity until exhaustion.

**Results:**

The results indicated that RIPC had no discernible effect on time to exhaustion compared to baseline or placebo conditions (*p* > 0.05). Moreover, parameters including MAOD_ALT_ (Alternative MAOD, derived from the fast component of excess post-exercise oxygen consumption and oxygen equivalent for blood lactate accumulation), lactic anaerobic capacity, alactic anaerobic capacity, and excess post-exercise oxygen uptake dynamics remained comparable across the three interventions (*p* > 0.05). Notably, a strong correlation was observed between MAOD and MAOD_ALT_ (*r* = 0.739; *p* < 0.05).

**Conclusion:**

In conclusion, this study provides evidence that remote ischemic preconditioning did not improve anaerobic capacity, as indicated by MAOD, among well-trained college tennis players. These findings emphasize the nuanced interplay of physiological factors in the context of RIPC and suggest that its impact on anaerobic capacity may be limited within this athletic cohort.

## Introduction

Tennis, an intermittent racket sport, traditionally thought to rely primarily on aerobic energy metabolism to sustain the energy demands required for optimal performance (Lees, 2003). However, the evolving nature of tennis towards an explosive sport, characterized by power, strength, and speed, has led to the prevalence of serves exceeding speeds of 210 km/h and average point durations in professional matches of less than 10 s (Kovacs, 2007). Besides, modern match-play is characterized by repeated high-intensity, short-duration efforts with brief recovery intervals, leading to an increased reliance on anaerobic metabolism. Intense and extended rallies, a hallmark of competitive tennis, necessitate the utilization of anaerobic glycolysis during frequent bursts of high-intensity efforts (Groppel & Roetert, 1992). The crucial role of anaerobic capacity in executing explosive groundstrokes and rapid directional changes underscores its significance for success in tennis (Kovacs, 2006). As a result, tennis athletes continuously explore strategies to enhance their anaerobic performance and maintain a competitive edge. One widely used index of anaerobic capacity is maximal accumulated oxygen deficit (MAOD), which quantifies the difference between oxygen demand and uptake during supramaximal exercise. Although valid, MAOD requires multiple submaximal exercise bouts and presents logistical challenges. To overcome this, an alternative single-test method (MAOD_ALT_) has been developed and validated in supramaximal contexts (Bertuzzi et al., 2010; Zagatto et al., 2016).

Remote ischemic preconditioning (RIPC) was originally introduced as a non-invasive intervention to protect organs against ischemia–reperfusion injury (Incognito, Burr & Millar, 2016), defined as tissue damage caused by the restoration of blood flow following a period of restricted circulation. RIPC involves brief cycles of blood flow occlusion and reperfusion applied to a limb, which may trigger systemic protective and metabolic adaptations. Initially developed to counteract the deleterious effects of ischemia-reperfusion injury (Murry, Jennings & Reimer, 1986), the precise mechanisms underlying RIPC’s actions remain subjects of ongoing investigation. It has been shown that RIPC improves metabolic efficiency by inhibiting ATP depletion and lactate production (Pang et al., 1995; Addison et al., 2003). Furthermore, evidence suggests that the effects of RIPC may involve intricate interactions within neuronal, humoral, and systemic responses (Caru et al., 2019). Building upon these findings, RIPC has shown promise as a viable preconditioning strategy when adapted for sports contexts (Incognito, Burr & Millar, 2016; Kilduff et al., 2013; Marocolo et al., 2019).

In previous studies, ischemic preconditioning (IPC), including RIPC, was theorized to enhance adenosine triphosphate (ATP) production through phosphogenic and glycolytic pathways—fundamental metabolic mechanisms closely tied to anaerobic capacity. A meta-analysis conducted in 2016 indicated that the effectiveness of IPC was consistent regardless of cuff placement location (*e.g*., local leg or remote arm), suggesting a plausible hypothesis that RIPC may potentiate exercise performance. Some studies reported improvements in MAOD—a widely recognized and arguably the most effective noninvasive marker of anaerobic capacity—as a result of RIPC interventions. Therefore, it seems reasonable to hypothesize that RIPC may potentiate exercise performance. However, investigations into the effects of RIPC on anaerobic capacity have yielded divergent outcomes. Others documented no significant enhancements in performance during various exercise tests, including the Wingate cycling test, 30-m sprints, and swimming time-trials. Furthermore, Patterson et al. (2015) noted that IPC had no discernible impact on blood lactate levels in their study.

In previous studies, ischemic preconditioning (IPC), including RIPC, was theorized to enhance adenosine triphosphate (ATP) production through phosphogenic (Andreas et al., 2011) and glycolytic (Janier, Vanoverschelde & Bergmann, 1994) pathways—fundamental metabolic mechanisms closely tied to anaerobic capacity (Green, 1995). A meta-analysis conducted in 2016 indicated that the effectiveness of IPC was consistent regardless of cuff placement location (*e.g*., local leg or remote arm) (Salvador et al., 2016), suggesting a plausible hypothesis that RIPC may potentiate exercise performance. Supporting this, some studies reported improvements in MAOD—a widely recognized noninvasive marker of anaerobic capacity (Medbø et al., 1988; Noordhof, de Koning & Foster, 2010; Cruz et al., 2016)—as a result of RIPC interventions (Chen et al., 2022; Paull & Van Guilder, 2019). These findings suggest that RIPC may enhance anaerobic performance under certain conditions.

However, investigations into the effects of RIPC on anaerobic capacity have also yielded null results. Several studies documented no significant improvements in performance across different exercise tests, including the Wingate cycling test, 30-m sprints, and swimming time trials (Lalonde & Curnier, 2015; Gibson et al., 2013; Williams et al., 2021). Furthermore, Patterson et al. (2015) reported that IPC had no discernible effect on blood lactate concentrations. Collectively, these findings indicate that RIPC does not consistently enhance performance, highlighting variability across exercise modalities and populations.

Despite growing interest in RIPC, limited research has examined its effects on intermittent, skill-based sports such as tennis. Understanding whether RIPC can enhance anaerobic capacity in well-trained tennis players is of practical interest for both performance optimization and recovery strategies. The present study aimed to investigate the effects of bilateral arm RIPC on supramaximal exercise performance, anaerobic capacity (MAOD, MAOD_ALT_), and metabolic responses in college tennis players. We hypothesized that RIPC can increase MAOD and MAOD_ALT_ during supramaximal workload exhaustion test.

## Materials and Methods

### Participants

Sixteen college well-trained tennis players (eight men and eight women) participated in the study. In addition to referencing prior studies, we conducted an *a priori* power analysis using G*Power 3.1 for repeated-measures ANOVA (within-subject, three conditions, two-tailed, α = 0.05). With *n* = 16, the design provides ≥80% power to detect medium-to-large within-subject effects (Cohen’s *f* = 0.25, corresponding to *ηp*^2^ ≈ 0.06). The participants had an average age of 20.9 ± 1.4 years, a mean height of 1.73 ± 0.76 m, a mean weight of 63.5 ± 8.2 kg, and a mean BMI of 21.2 ± 1.4 kg/m^2^, and a mean VO_2max_ of 50 ± 6 ml/min/kg (Table 1). All participants were actively competing at collegiate level, trained at least 10–12 h per week, they were selected from the elite range of international tennis players based on their International Tennis Numbers (ITNs) and held either first-class or second-class national rankings. The criterion for “well-trained” was based on these benchmarks in addition to a VO_2max_ above 50 ml/min/kg. The sample size was determined based on the expected effect of RIPC on performance and MAOD change, as reported in previous studies (Chen et al., 2023; Paull & Van Guilder, 2019). Inclusion criteria for the participants were as follows: (1) no history of smoking, (2) no prior diagnosis of hypertension, cardiovascular, pulmonary, or metabolic illnesses, (3) no back or musculoskeletal injuries in the past three months. Before the experiments began, each participant received detailed information about the experimental methods and procedures, and signed a written informed consent. These procedures were demonstrated by one of the researchers. However, the participants were not further counseled to prevent the experimental procedure from becoming an active component of the intervention. The study was conducted in accordance with the principles outlined in the Helsinki Declaration and was approved by the Ethics Committee for Human Studies of the Physical Education Department of North China University of Water Resources and Electric Power (20240501046).

**Table 1:** Athlete participant characteristics.

Variable	Total group (*n* = 16)
Sex, men/women	8/8
Age, year	20.9 ± 1.4
Height, m	1.73 ± 0.76
Weight, kg	63.5 ± 8.2
BMI, kg/m^2^	21.2 ± 1.4
Percent fat, %	19.3 ± 5.9
Muscle mass, kg	49.8 ± 8.7
VO_2max_, ml/min/kg	50 ± 6
HR_max_, bpm	195 ± 12
Training experience, year	7.9 ± 2.4
ITN 1/2	8/8

### Experimental design

A randomized, counterbalanced crossover design was employed. This study comprised five separate laboratory exercise sessions: one maximal incremental running test, one constant workload test at submaximal intensities (40–80% VO_2max_), and three supramaximal effort tests (110% VO_2max_). Each participant completed three conditions: Baseline (no intervention), Placebo (cuffs applied without inflation), and RIPC (cuffs inflated). Sessions were separated by at least 72 h to minimize fatigue and carryover. The rationale for this design was that Baseline minimized learning effects, Placebo controlled for expectancy, and RIPC assessed intervention effects. The study was conducted in a single-blind manner: the experimenter collecting and analyzing data was blinded to condition, while participants were unaware of the hypothesis but may have perceived cuff inflation intensity.

Before each laboratory visit, an outdoor International Tennis Number (ITN) test was conducted to determine the training status of the participants. Initially, participants established their baseline MAOD values by undergoing a supramaximal test. Following this baseline assessment, participants were randomly assigned to either the RIPC group or the Placebo group (involving a supramaximal effort run lasting ≥2 min).

The randomization sequences were generated using a tool called research randomizer (https://www.randomizer.org/) and were enclosed within opaque, consecutively numbered envelopes by an independent researcher to maintain concealment. A minimum of 72 h separated the supramaximal effort tests (Chen et al., 2022; Paull & Van Guilder, 2019). Throughout all experiments, consistent environmental conditions were maintained (temperature: 21.9 ± 1.4 °C, relative humidity: 46.5 ± 4.8%). To mitigate the potential impact of circadian rhythms on exercise performance and other outcomes (Atkinson & Reilly, 1996), the experiments were conducted at the same time of day.

### Remote ischemic preconditioning

**Figure 1:**
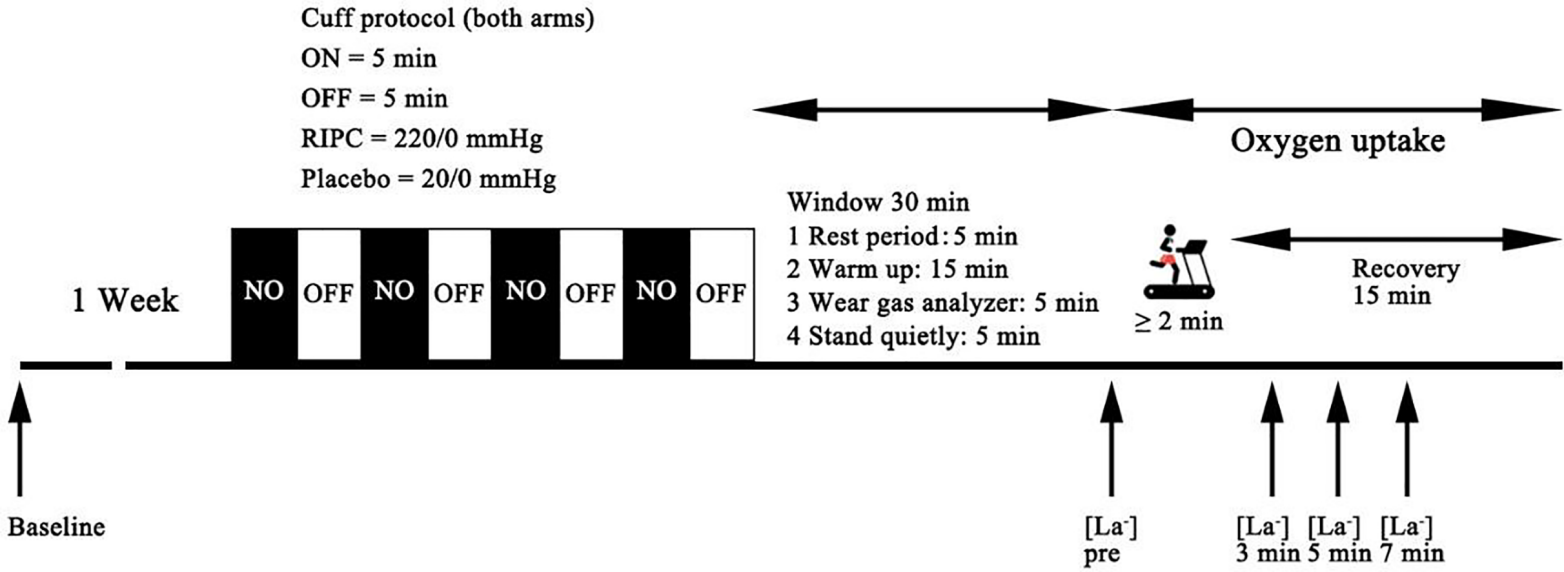
Schematic representation of the experimental protocol of the present study.

Following the establishment of baseline measurements, the participants were randomly divided into either the RIPC group or the placebo group. The subsequent running tests took place immediately after the baseline measurements, with a minimum interval of 1 week between sessions (Fig. 1).

For the RIPC intervention, a specific procedure was followed. All participants assumed a supine position, and automatic occlusion cuff belts (52 mm wide) were securely positioned on their arms using a device known as the Kaatsu Master (Kaatsu Master, Sato Sports Plaza, Tokyo, Japan). The cuffs were intended to occlude the brachial artery for a period of 5 min, temporarily restricting blood flow to the arms. This occlusion phase was followed by a 5-min reperfusion period, during which the cuffs were deflated, allowing blood to flow back into the arms. The RIPC intervention was completed 30 min before the supramaximal effort test. The cuff pressure in the experimental group was 220 mmHg, while that in the placebo group was 20 mmHg. The pressure was automatically monitored by the equipment and maintained at the set value. This alternating cycle of occlusion and reperfusion was repeated a total of four additional times, consistent with established RIPC protocols that have been previously utilized in other exercise performance trials (Caru et al., 2019; Salvador et al., 2016). Conversely, the Placebo trial closely resembled the RIPC procedure, but with a key difference. In this case, the pressure cuffs were inflated to a lower pressure of 20 mmHg, a level that would not induce significant ischemia (blood flow restriction) or subsequent reperfusion in most individuals, though individual variations cannot be ruled out. This ensured that the Placebo group did not experience the preconditioning effects of RIPC, and we also tested the baseline values of the subjects as a control group that did not undergo any ischemic intervention.

### Outcome measures

#### Maximal incremental exercise test

The maximal incremental exercise test was conducted on a treadmill (Stratos med; H/p/cosmos, Nussdorf-Traunstein, Germany) with a 1% incline, as outlined in the study (Chen et al., 2022). Breath-by-breath measurement of oxygen uptake was performed using an oxygen analyzer (MetaMax 3B, Cortex Biophysics, Leipzig, Germany), with data recorded at 10-s intervals. The calibration of the gas analyzers was carried out according to the instructions provided in the MetaMax 3B instruction manual, utilizing calibration gas and an air volume syringe containing 25% oxygen and 5% carbon dioxide. Additionally, a blood sample (25 µl) was collected from the participant’s finger immediately after completing the exercise. The measurement of blood lactate accumulation was conducted using a Biosen S-line blood lactate analyzer (EKF Diagnostic, Barleben, Germany).

Prior to commencing the maximal incremental test, participants underwent a standardized warm-up procedure, involving a 5-min running period at 8 km/h and a 5-min stretching routine. The test itself began with an initial running velocity of 10 km/h, with subsequent increments of 1 km/h every 2 min until the point of voluntary exhaustion was reached. Participants received strong verbal encouragement throughout the test, motivating them to continue running for as long as possible.

The criterion for determining the achievement of VO_2max_, as well as the minimum velocity at which VO_2max_ was reached, involved consideration of specific factors as follows (Andrade et al., 2020; Bertuzzi et al., 2010): (1) a VO_2_ increase of less than 2.1 ml/kg/min across two consecutive stages, (2) blood lactate concentrations exceeding 8.0 mmol/L, (3) respiratory exchange ratio (RER) of 1.10 or higher, (4) Heart rate within ±10 bpm of the maximum heart rate predicted by age (220-age). VO_2max_ was defined as the highest mean VO_2_ recorded during the last 30 s of the test, taking into account the above criteria. This comprehensive approach ensured accurate determination of participants’ maximal oxygen uptake and the associated minimum velocity.

#### Submaximal workload tests

Submaximal workload tests were conducted on the treadmill under conditions identical to those of the maximal incremental test. These tests involved varying exercise intensities, specifically corresponding to 40%, 50%, 60%, 70%, and 80% of each participant’s individual VO_2max_ values.

During each test, participants exercised at the specified intensity until a consistent and stable oxygen consumption (VO_2_) value was attained over a duration of at least 120 s. During the exercise sessions, participants were randomly assigned to complete 1 to 2 of these submaximal workload tests at different intensities. After completing each submaximal workload test, a recovery period of 10 min was observed before the next test or until the participant’s oxygen consumption (VO_2_) returned to a baseline level (Bertuzzi et al., 2010). This recovery period ensured that participants were adequately rested and stabilized before proceeding to the next test.

#### Supramaximal effort test

After completing both the RIPC and placebo trials, participants were readied for the supramaximal effort test, which aimed to measure the MAOD. This test involved performing exercise at a workload equivalent to 110% of each participant’s individual VO_2max_.

To initiate the supramaximal effort test, participants engaged in a 15-min warm-up session by running at an average speed of 13.7 km/h (Paull & Van Guilder, 2019). While the optimal rest duration between the final RIPC cycle and the commencement of exercise has yet to be established, existing meta-analysis data suggest potential benefits to anaerobic performance when RIPC is concluded at least 30 min before exercise initiation (Salvador et al., 2016).

To establish a baseline for oxygen consumption (VO_2_), participants stood quietly on a treadmill for 5 min before initiating the supramaximal exhaustion test. Blood samples were collected from each participant’s finger immediately before exercise, as well as at 3, 5, and 7 min post-exercise cessation to measure peak blood lactate levels. Throughout the running test and for an additional 15 min post-test, participants wore masks to facilitate the collection of accurate VO_2_ data.

#### Calculations of the MAOD and MAOD_ALT_

As outlined by Medbø et al. (1988), the MAOD is defined as the disparity between the anticipated oxygen demand and the actual oxygen consumption over the course of a maximal exercise session. The theoretical oxygen uptake during supramaximal exercise was derived using a linear regression equation, which incorporated VO_2_ measurements and relative intensities obtained from five submaximal running trials to establish the VO_2_-velocity regression model (mean *R*^2^ = 0.95 ± 0.02). During the execution of the supramaximal test, a breath-by-breath assessment of VO_2_ was conducted to accurately determine the actual cumulative oxygen uptake. Consequently, the expression for MAOD is formulated as follows: MAOD (ml/kg) = theoretical accumulated oxygen demand (ml/min/kg) × running duration (min) − actual accumulated oxygen uptake (ml/min/kg) × running duration (min).

**Figure 2:**
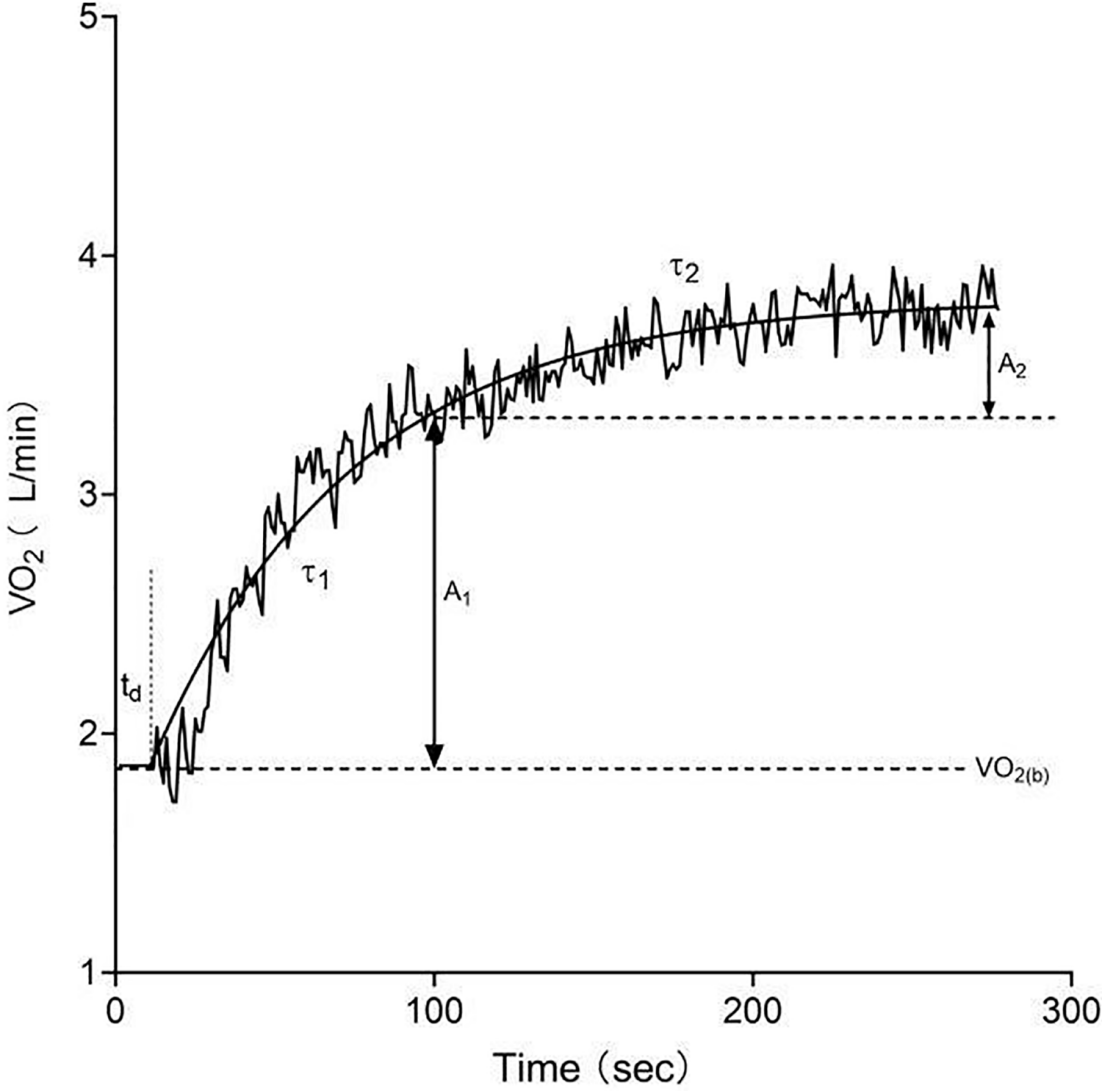
Oxygen uptake kinetics during exercise modeled with a bi-exponential function. A_1_, asymptotic value of the exponential term in the fast response phase; t_d_, time delay; τ_1_, time constant of the fast response phase; A_2_, asymptotic value of the exponential term in the slow response phase; τ_2_, time constant of the slow response phase.

MAOD_ALT_ is determined through the summation of the alactic (phosphagen metabolic pathway) and lactic anaerobic (glycolytic metabolic pathway) anaerobic metabolisms. The quantification of the alactic anaerobic metabolism contribution to MAOD_ALT_ involved the application of the EPOC_FAST_ method (Bertuzzi et al., 2010; Margaria, Edwards & Dill, 1933), which was computed using VO_2_ data collected over a 15-min recovery period. To predict the breath-to-breath VO_2_ off-transients during the supramaximal exercise test, a biexponential model was employed (Eq. (1)) and fitted using GraphPad Prism 8.0 software (GraphPad Software, San Diego, CA, USA) (Bertuzzi et al., 2010). The process of refining the oxygen uptake data adhered to established protocols (Bertuzzi et al., 2016), encompassing the stabilization of the VO_2_ baseline and the application of a mono-exponential fit to achieve bi-exponential estimates (Fig. 2). Ultimately, EPOC_FAST_ was determined as the product of *A*_1_ and τ_1_.

(1) $${\rm VO}_{2(t)} = {\rm VO}_{2(b)} + A_1 \times e^{-(t -t_{d})/\tau_1} + A_2 \times e^{-(t - t_{d})/{\tau_2}}$$

(2) $$EPOC_{FAST} = {A_1} \times {\tau _1}.$$

In the aforementioned equation, VO_2(*t*)_ represents the oxygen uptake at time *t*, VO_2_ baseline denotes the baseline oxygen uptake, *A* signifies the amplitude, *t_d_* stands for time delay, and τ indicates the time constant. The subscripts 1 and 2 correspond to the fast and slow components, respectively. Curve fits with *R*^2^ < 0.9 were to be rejected and re-checked; all final models achieved *R*^2^ ≥ 0.90. The lactic anaerobic contribution (ml/kg) arising from blood lactate was determined based on the accumulation of blood lactate during the supramaximal exercise tests, applying an assumption of a 3.0 ml/kg/mmol·L oxygen-lactate equivalent (Di Prampero, 1981).

### Statistical analysis

Statistical analyses were carried out using SPSS version 24.0 (IBM Inc., Armonk, NY, USA). The pre-specified primary outcome was MAOD (and time to exhaustion); secondary outcomes included MAOD_ALT_, anaerobic alactic and lactic contributions, and VO_2_ kinetics parameters. Prior to analysis, a Shapiro-Wilk test was conducted to assess the distribution of data, confirming a normal Gaussian distribution. Unless explicitly stated, all data are presented as means ± standard deviation (SD). Repeated-measures ANOVA was used to assess differences across conditions. Mauchly’s test of sphericity was performed, and when violated, Greenhouse–Geisser corrections were applied. *Post hoc* pairwise comparisons with Bonferroni adjustment were conducted where appropriate. Effect sizes are reported as partial eta squared (*ηp*^2^) for ANOVA main effects and Cohen’s *dz* for pairwise contrasts, together with 95% confidence intervals. The paired t-test was utilized to assess differences between MAOD and MAOD_ALT_. Additionally, Pearson correlations were employed to compare MAOD and MAOD_ALT_, and the agreement between the two was assessed through Bland-Altman plots. All raw data, including individual-level values for time to exhaustion, oxygen uptake, blood lactate, MAOD, and MAOD_ALT_, were preserved and analyzed without exclusion. Statistical significance was set at a significance level of 0.05.

## Results

### Primary outcome measures

Time to exhaustion did not differ significantly across conditions (Baseline: 208.31 ± 69.54 s; Placebo: 202.81 ± 63.24 s; RIPC: 207.25 ± 61.63 s; *F*(2, 30) = 0.145, *p* = 0.866, *ηp*^2^ = 0.010; data shown in Table 2). Pairwise comparisons confirmed trivial effects: Placebo *vs*. Baseline (mean diff = –5.5 s, 95% CI [–31.1 to 20.1], *dz* = –0.11), RIPC *vs*. Baseline (–1.1 s, 95% CI [–25.4 to 23.3], *dz* = –0.02), and RIPC *vs*. Placebo (+4.4 s, 95% CI [–14.5 to 23.4], *dz* = 0.12).

**Table 2:** Results for the supramaximal effort test.

	Baseline (*n* = 16)	Placebo (*n* = 16)	RIPC (*n* = 16)	Mauchly’s test of sphericity	*df*	*F*	*p*-value	*ηp* ^*2*^
Time to exhaustion (s)	208.31 ± 69.54	202.81 ± 63.24	207.25 ± 61.63	*W* = 0.886, *p* = 0.430	2	0.145	0.866	0.010
MAOD (ml/kg)	49.70 ± 10.11	49.70 ± 12.05	50.69 ± 14.00	*W* = 0.946, *p* = 0.680	2	0.103	0.903	0.007
MAOD_ALT_ (ml/kg)	51.06 ± 6.48	51.14 ± 7.42	51.33 ± 8.40	*W* = 0.922, *p* = 0.566	2	0.009	0.991	0.001
Anaerobic alactic (ml/kg)	21.83 ± 5.17	22.94 ± 5.48	22.37 ± 6.59	*W* = 0.743, *p* = 0.125	2	0.235	0.792	0.015
Anaerobic lactic (ml/kg)	29.24 ± 6.91	28.20 ± 5.70	28.96 ± 5.64	*W* = 0.523, *p* = 0.011	1.354	0.297	0.660	0.019

### Anaerobic capacity (MAOD and MAOD_ALT_)

No significant condition effects were observed for MAOD (Baseline: 49.70 ± 10.11 ml·kg^−1^; Placebo: 49.70 ± 12.05 ml·kg^−1^; RIPC: 50.69 ± 14.00 ml·kg^−1^; *F*(2, 30) = 0.103, *p* = 0.903, *ηp*^2^ = 0.007; Table 2). Pairwise comparisons showed negligible differences: Placebo *vs*. Baseline (mean diff = 0.0, 95% CI [–5.1 to 5.1], *dz* = 0.00), RIPC *vs*. Baseline (+1.0, 95% CI [–4.0 to 6.0], *dz* = 0.11), and RIPC *vs*. Placebo (+1.0, 95% CI [–5.0 to 6.9], *dz* = 0.09).

Similarly, MAOD_ALT_ values were nearly identical across trials (Baseline: 51.06 ± 6.48 ml·kg^−1^; Placebo: 51.14 ± 7.42 ml·kg^−1^; RIPC: 51.33 ± 8.40 ml·kg^−1^; *F*(2, 30) = 0.009, *p* = 0.991, *ηp*^2^ = 0.001; Table 2). Pairwise contrasts indicated trivial effects: Placebo *vs*. Baseline (+0.07, 95% CI [–4.3 to 4.5], *dz* = 0.01), RIPC *vs*. Baseline (+0.27, 95% CI [–3.4 to 4.0], *dz* = 0.04), and RIPC *vs*. Placebo (+0.19, 95% CI [–4.6 to 5.0], *dz* = 0.02).

Anaerobic alactic contributions (Baseline: 21.83 ± 5.17 ml·kg^−1^; Placebo: 22.94 ± 5.48 ml·kg^−1^; RIPC: 22.37 ± 6.59 ml·kg^−1^; *F*(2, 30) = 0.235, *p* = 0.792, *ηp*^2^ = 0.015; Table 2) and lactic contributions (Baseline: 29.24 ± 6.91 ml·kg^−1^; Placebo: 28.20 ± 5.57 ml·kg^−1^; RIPC: 28.96 ± 5.64 ml·kg^−1^; *F*(2, 30) = 0.297, *p* = 0.660, *ηp*^2^ = 0.019; Table 2) also showed no significant pairwise differences (all *dz* < 0.20, *CI*s overlapping zero).

**Figure 3:**
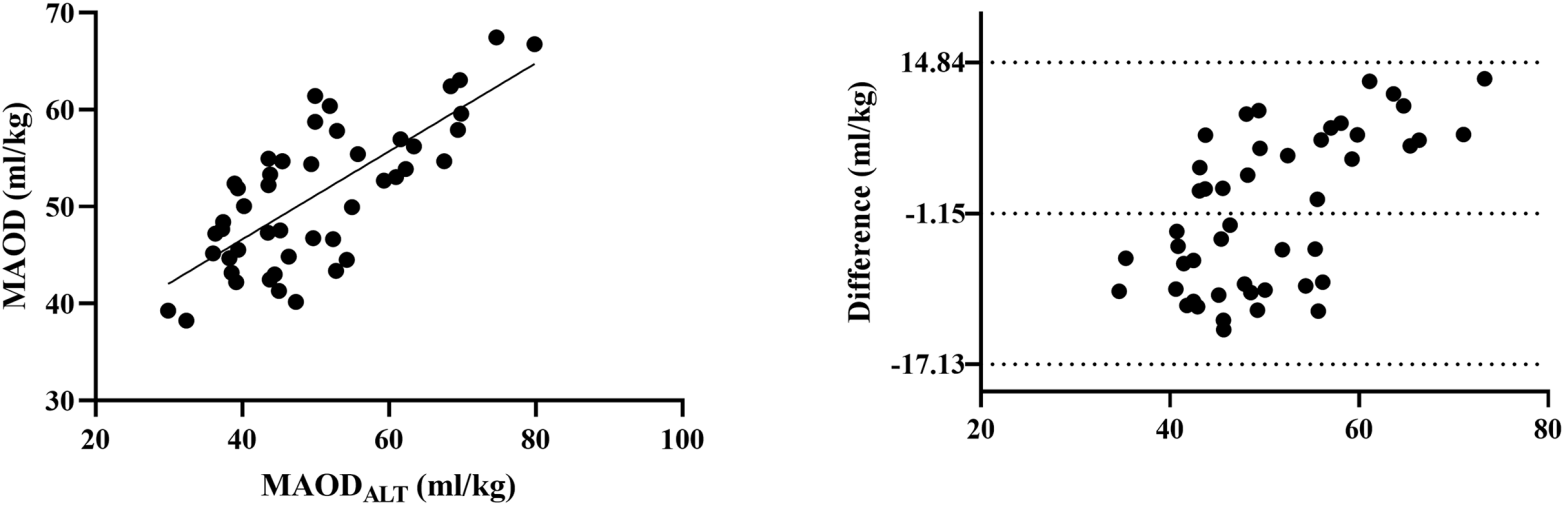
Comparison between MAOD and MAOD_ALT_. Left: Pearson correlation graph for visualizing the relationship between MAOD and MAOD_ALT_. Right: Bland-Altman graph for visualizing the difference between MAOD and MAOD_ALT_.

Importantly, MAOD and MAOD_ALT_ were strongly correlated across all participants (*r* = 0.739, *p* < 0.001), reinforcing the validity of MAOD_ALT_ as a surrogate for anaerobic capacity (see Fig. 3).

### Oxygen uptake kinetics

Parameters describing oxygen uptake kinetics (*A*_1_, *td, τ*_1_, *A*_2_, *τ*_2_, and *R*^2^) were comparable among Baseline, Placebo, and RIPC conditions (all *p* > 0.05, *ηp*^2^ < 0.07; data summarized in Table 3). For example, mean *A*_1_ values were 1.45 ± 0.21, 1.46 ± 0.20, and 1.41 ± 0.26 l·min^−1^, respectively (*F*(1.354, 20.31) = 0.720, *p* = 0.447, *ηp*^2^ = 0.046). Time delay (*t*_*d*_) was 25.21 ± 7.56, 25.49 ± 7.02, and 25.45 ± 7.94 s (*F*(2, 30) = 0.053, *p* = 0.949, *ηp*^2^ = 0.003). The primary time constant *τ*_1_ was 57.13 ± 9.60, 59.51 ± 10.98, and 60.35 ± 16.10 s (*F*(1.422, 21.33) = 0.309, *p* = 0.663, *ηp*^2^ = 0.020). *A*_2_ was 0.45 ± 0.21, 0.48 ± 0.20, and 0.49 ± 0.22 l·min^−1^ (*F*(1.454, 21.81) = 0.239, *p* = 0.718, *ηp*^2^ = 0.016). The slow component time constant *τ*_2_ was 414.17 ± 188.82, 411.76 ± 77.88, and 389.97 ± 170.78 s (*F*(2, 30) = 0.094, *p* = 0.910, *ηp*^2^ = 0.006). Goodness of fit (*R*^2^) was high in all conditions (0.92 ± 0.04, 0.90 ± 0.05, 0.90 ± 0.05; *F*(2, 30) = 1.061, *p* = 0.359, *ηp*^2^ = 0.066) data summarized in Table 3.

**Table 3:** Results of kinetics parameters based on bi-exponential model of EPOC.

Parameter	Baseline (*n* = 16)	Placebo (*n* = 16)	RIPC (*n* = 16)	Mauchly’s test of sphericity	*df*	*F*	*p*-value	*ηp* ^2^
A_1_ (l/min)	1.45 ± 0.21	1.46 ± 0.20	1.41 ± 0.26	*W* = 0.523, *p* = 0.011	1.354	0.720	0.447	0.046
t_d_ (s)	25.21 ± 7.56	25.49 ± 7.02	25.45 ± 7.94	*W* = 0.720, *p* = 0.101	2	0.053	0.949	0.003
τ_1_	57.13 ± 9.60	59.51 ± 10.98	60.35 ± 16.10	*W* = 0.594, *p* = 0.026	1.422	0.309	0.663	0.020
A_2_ (l/min)	0.45 ± 0.21	0.48 ± 0.20	0.49 ± 0.22	*W* = 0.625, *p* = 0.037	1.454	0.239	0.718	0.016
τ_2_	414.17 ± 188.82	411.76 ± 77.88	389.97 ± 170.78	*W* = 1, *p* = 0.999	2	0.094	0.910	0.006
R^2^	0.92 ± 0.04	0.90 ± 0.05	0.90 ± 0.05	*W* = 0.994, *p* = 0.957	2	1.061	0.359	0.066

**Notes:**

A_1_: Asymptotic value of the exponential term in the fast response phase.

t_d_: Time delay.

τ_1_: Time constant of the fast response phase.

A_2_: Asymptotic value of the exponential term in the slow response phase.

τ_2_: Time constant of the slow response phase.

R^2^: Goodness of fit of the model.

Collectively, these results (summarized in Tables 2 and 3) demonstrate that RIPC did not significantly alter time to exhaustion, anaerobic capacity indices, or oxygen uptake kinetics during supramaximal exercise.

## Discussion

The present study aimed to investigate the effects of bilateral arm RIPC on supramaximal exercise performance and metabolic responses in well-trained college tennis players. The main finding was that RIPC had no significant influence on time to exhaustion, MAOD, anaerobic alactic and lactic contributions, or oxygen uptake and extraction kinetics during a supramaximal treadmill test. All indices of anaerobic capacity (MAOD, MAOD_ALT_, alactic and lactic components) and oxygen uptake kinetics were highly comparable across Baseline, Placebo, and RIPC trials. Effect sizes for pairwise comparisons were trivial, indicating that any true ergogenic effect of the present RIPC protocol on these outcomes is likely to be small at best.

From a mechanistic perspective, the absence of measurable benefits may reflect prior physiological adaptations in this well-trained cohort that limit the scope for further improvement *via* RIPC. Regular high-intensity training, as undertaken by the current participants, is known to enhance endothelial function, skeletal muscle oxidative capacity, and metabolic regulation, and may itself induce ischemic-preconditioning-like adaptations (Crisafulli, 2006). These adaptations include improved microvascular function, greater ischemic tolerance, and more efficient matching of O_2_ delivery to utilization, which can reduce ATP depletion and lactate accumulation during intense exercise (Incognito, Burr & Millar, 2016; Pang et al., 1995; Addison et al., 2003). In such a context, additional protection or metabolic “fine-tuning” elicited by RIPC may be redundant or too small to detect with our outcome measures. This ceiling effect hypothesis is consistent with work suggesting that the performance gains achievable by ergogenic interventions become progressively smaller as baseline fitness increases (Hopkins, Hawley & Burke, 1999).

Inter-individual variation may further contribute to the heterogeneous findings in the literature. It is well established that responses to therapeutic and exercise interventions vary widely among individuals (Incognito, Burr & Millar, 2016; Bouchard & Rankinen, 2001). Although we did not pre-specify or statistically model “responders” and “non-responders,” visual inspection of individual data suggested that some players exhibited small improvements in supramaximal tolerance following RIPC, whereas others remained essentially unchanged. Our study was not powered or designed to classify responders, and these observations must therefore be interpreted cautiously. Nonetheless, they align with the concept that both responders and non-responders may coexist within a given intervention group (Caru et al., 2019) and highlight the need for future work to use designs and sample sizes that allow formal characterization of individual response patterns, including potential genetic or phenotypic determinants.

Training status and sport-specific fitness profiles may also help reconcile the divergent effects of RIPC reported across studies. In Paull & Van Guilder (2019) and Chen et al. (2023), RIPC increased MAOD and supramaximal performance in moderately trained middle-distance and 400 m runners. In contrast, the present participants were well-trained tennis players with a mean VO_2max_ of ~50 ml/min/kg, lower than the ~65 and ~58 ml/min/kg reported in those running cohorts. Differences in aerobic capacity, anaerobic capacity, and muscle phenotype between endurance-oriented runners and intermittent racket-sport athletes may influence both the magnitude and mechanisms of any RIPC effect. Moreover, exercise itself can confer ischemic preconditioning-like myocardial and skeletal muscle protection (Crisafulli, 2006), and Tocco et al. (2015) suggested that long-term high-intensity training may blunt the additional benefits of experimental IPC. Thus, the combination of sport-specific training background and already well-developed cardiovascular–metabolic adaptations in our players may have reduced the window for detectable ergogenic gains.

A central interpretative issue is the suitability of our experimental model for the demands of tennis. Tennis is characterized by repeated, short-duration, high-intensity efforts interspersed with brief recovery intervals and complex technical and tactical actions (Kovacs, 2006). In contrast, our primary outcomes were derived from a continuous supramaximal treadmill run to exhaustion and the associated MAOD and MAOD_ALT_ calculations. These tests are well established for quantifying anaerobic capacity in laboratory settings (Noordhof, de Koning & Foster, 2010; Medbø et al., 1988; Bertuzzi et al., 2010), but they primarily reflect the capacity to sustain a single exhaustive bout rather than the intermittent, multidirectional, and skill-dependent efforts typical of tennis. It is therefore plausible that the null result observed here partly reflects a measurement mismatch: RIPC may have limited or context-specific effects on repeated sprint ability, change-of-direction performance, or between-point recovery that are not captured by continuous running MAOD tests. Future studies should address this by integrating tennis-specific performance measures (*e.g*., repeated shuttle sprints, agility tests, point-simulation protocols) alongside physiological indices.

The comparison with earlier studies also raises the question of local *versus* remote application and the muscle groups engaged. While meta-analytic data suggest that IPC can exert systemic effects regardless of cuff placement (Salvador et al., 2016), more recent work indicates that local ischemic preconditioning applied directly to the working musculature may elicit stronger ergogenic responses than remote protocols. Griffin et al. (2018) reported that leg IPC sometimes produced more pronounced benefits on repeated sprint running performance than arm RIPC, and Weerapong et al. (2025) observed modality-dependent differences in muscle deoxygenation kinetics between local and remote IPC during a 3-min all-out cycling test. Similarly, Allois et al. (2025) emphasized that using small-muscle-group models helps to isolate local IPC mechanisms and minimize systemic confounding, and that not all remote effects are reliably transferred. In our study, RIPC was applied to the arms, whereas the supramaximal task primarily stressed the lower limbs. The absence of measurable benefit therefore may not solely indicate that RIPC is ineffective, but rather that upper-limb RIPC exerts limited systemic influence on lower-limb performance in this population and test model.

With respect to anaerobic metabolism, both MAOD and MAOD_ALT_ remained unchanged across conditions. Previous studies suggested that RIPC might enhance intramuscular phosphocreatine availability, modulate glycolytic flux, or improve buffering capacity, thereby influencing the balance between alactic and lactic energy contributions (Murry, Jennings & Reimer, 1986; Caru et al., 2019; Pang et al., 1995; Bertuzzi et al., 2010). However, blood lactate is determined by both production and clearance (Bertuzzi et al., 2016), and stable lactate and MAOD values in the present study indicate that, under our protocol, RIPC did not meaningfully alter the overall anaerobic energy yield or the lactate production—removal equilibrium during supramaximal exercise. The strong correlation between MAOD and MAOD_ALT_ observed across trials supports the validity of MAOD_ALT_ as a practical estimator of anaerobic capacity, but also underscores that both indices converged on the same conclusion: bilateral arm RIPC did not measurably enhance anaerobic energy contribution in this cohort.

Methodological considerations, particularly regarding placebo control and blinding, must also be acknowledged. The distinct discomfort associated with high-pressure limb occlusion makes it difficult to create a truly indistinguishable sham condition (Lalonde & Curnier, 2015). Although we used standardized external pressures and single-blind procedures, participants could plausibly distinguish between high-pressure RIPC and low-pressure placebo, which may have influenced expectations. Employing sham pressures just below the ischemic threshold and explicitly assessing perceived pressure and pain may strengthen blinding in future work. Nonetheless, given the trivial effect sizes and highly overlapping confidence intervals across conditions, it seems unlikely that imperfect blinding alone masked a large, practically relevant ergogenic effect in the present study.

Finally, several limitations should be considered when interpreting these findings. The sample size was modest (*n* = 16), which limits the ability to detect very small effects and to undertake subgroup analyses (*e.g*., by sex, fitness level, or putative responder status). The single-blind design, incomplete participant blinding, and reliance on a laboratory treadmill protocol further constrain generalizability to on-court performance. Exclusion criteria were relatively broad, and more stringent characterization of training history, playing style, and muscle function may help identify subgroups more likely to benefit from RIPC. Addressing these limitations in future studies—through larger samples, rigorous double-blind designs, tennis-specific performance tests, and comparison of local *versus* remote IPC—will be important for clarifying the extent to which ischemic preconditioning can influence performance in intermittent, high-intensity racket sports.

## Conclusions

This study found that upper-limb remote ischemic preconditioning, applied as four cycles of 5-min bilateral arm occlusion at 220 mmHg, did not improve supramaximal running performance or anaerobic capacity markers (MAOD, MAOD_ALT_, alactic and lactic contributions) in well-trained college tennis players. Within the context of a continuous supramaximal treadmill test, the protocol produced trivial and non-significant changes in time to exhaustion, metabolic responses, and oxygen uptake kinetics compared with baseline and placebo trials. These findings should be interpreted in light of the specific population and experimental model used. The participants were already well trained, and the primary outcome reflected tolerance to a single exhaustive bout of running rather than the repeated, skill-dependent efforts typical of tennis. As such, the results indicate that, under these conditions, upper-limb RIPC provides no measurable ergogenic benefit on laboratory-based indices of anaerobic capacity in this athlete group. Future investigations should prioritize sport-specific, intermittent performance tests that better reflect tennis demands (*e.g*., repeated-sprint ability, change-of-direction tasks, and between-point recovery) and consider local ischemic preconditioning applied to the lower limbs. Standardizing RIPC protocols and more precisely characterizing athlete training status will be important for determining whether particular applications, populations, or test modalities can yield meaningful performance improvements.

## Supplemental Information

10.7717/peerj.20789/supp-1 Supplemental Information 1 Raw data.

## Additional Information and Declarations

### Data Availability

The following information was supplied regarding data availability:

The raw data are available in the Supplemental File.
